# Prevalence and predictors of use of mental health and well-being support among healthcare workers in England: findings from NHS CHECK

**DOI:** 10.1192/bjo.2026.12108

**Published:** 2026-10-09

**Authors:** Brendan Dempsey, Pamela Almeida-Meza, Bethany Croak, Danielle Lamb, Neil Greenberg, Ira Madan, Rosalind Raine, Anne Marie Rafferty, Rupa Bhundia, Simon Wessely, Sharon A. M. Stevelink

**Affiliations:** Department of Psychological Medicine, Institute of Psychiatry, Psychology & Neuroscience, King’s College London, UK; Department of Primary Care and Population Health, University College London, UK; Guy’s and St Thomas’ Hospitals NHS Foundation Trust, London, UK; Florence Nightingale Faculty of Nursing, Midwifery and Palliative Care, King’s College London, UK

**Keywords:** Longitudinal data, multilevel modelling, psychological treatments, healthcare workers, mental health services

## Abstract

**Background:**

Healthcare can be a demanding career, and there is a rising interest in fostering the well-being of healthcare workers (HCWs).

**Aims:**

To examine self-reported use of mental health and well-being support available to HCWs and explore predictors of use.

**Method:**

Data were collected by a longitudinal cohort study. The baseline survey was distributed from April 2020, followed by three surveys at 6 months (time 1; *n* = 10 514), 12 months (time 2; *n* = 10 429) and 32 months (time 3; *n* = 6382) post baseline. Use of mental health support (e.g. therapy) was assessed at each follow-up, and 96 supports were measured at time 1 only. Multilevel regression modelling was used to explore baseline predictors.

**Results:**

At time 1, we developed seven support categories. Practical supports (e.g. free or discounted food or parking) were the most commonly used (58%), whereas employee assistance programmes were least commonly used (5%). Furthermore, 21% of HCWs reported never using any support. Mental health support access varied: 23% of participants had used support before August 2021 (time 1), 17% between March 2020 and January 2022 (time 2), and 21% between February 2022 and May 2023 (time 3). HCWs who were male, older and/or of Asian ethnicity had lower odds of accessing support, whereas those who reported symptoms of common mental disorders at baseline had higher odds.

**Conclusions:**

Most HCWs (79%) reported having accessed some support, whereas 21% had not. Practical and informal supports were most used, whereas uptake of employee assistance programmes was low. Future research should explore and address barriers faced by male, older, Asian and non-permanent staff to improve access and, in turn, the well-being, performance and patient care of these individuals.

Healthcare systems worldwide have long struggled to recruit and retain enough staff,^1,2^ and mental ill health is a leading reason for poor retention of healthcare workers (HCWs).^3^ An analysis of National Health Service (NHS) digital data found that mental ill health was the leading reported cause of sickness absence in the first half of 2024 in the UK, accounting for approximately 24–27% of all NHS staff absences per month.^4^ By comparison, the Office for National Statistics estimated that mental health was the third leading cause of absence among all UK workers in 2022, accounting for 7.9% of absences.^5^ Presenteeism, or attending work when unwell and not functioning at full capacity,^6^ is also common among HCWs experiencing mental ill health.^7^ A recent umbrella review of 87 meta-analyses estimated that 30% of worldwide HCWs may experience symptoms of anxiety, and that 28% have symptoms of depression.^8^ However, symptoms are not disorders, and the true prevalence of anxiety or depressive disorders, often referred to as common mental disorders (CMDs), is lower. We previously found that although 52.8% of HCWs in a nationally representative sample screened positive for symptoms of CMDs, 21.5% met the clinical definition for a CMD following diagnostic assessment.^9^ Occupational factors in healthcare that may contribute to CMDs include long shifts, large workloads, lack of physical and psychological safety, moral injury, caring for sick patients, workplace bullying and discrimination, and lack of social support.^3^ The COVID-19 pandemic has contributed to increased estimates of CMDs among adults in the UK.^10^ In addition to affecting HCWs and their families, poorer well-being of HCWs is associated with worse patient outcomes.^11^ The pandemic brought this issue to the foreground, as the public relied heavily on HCWs to care for those who became sick.

The foremost forms of support for people experiencing mental ill health in the UK are talking therapies approved by the National Institute for Health and Care Excellence, counselling and psychotropic medications. These can be accessed through the workplace or elsewhere, such as via a general practitioner. Such interventions can be administered face to face or virtually and have proven effective in improving the mental health of HCWs and alleviating stress caused by workplace demands.^12,13^ They have also been shown to be effective in supporting people to return to work faster after sickness absence,^14^ although we have not seen this evidence reviewed among HCWs specifically. Despite the availability and potential benefits of such supports, many HCWs do not access them, and there are known barriers to help-seeking; these include stigma, negative career implications, hostile work environments, lack of awareness of services, lack of availability of services, and lack of time in which to seek help.^15,16^ Further, a review of population-based cohorts found that people who were male, Black and minority ethnic, married or did not have higher education had lower odds of utilising health services for CMDs.^17^ Understanding the factors that influence help-seeking behaviours in an NHS context could help us to identify groups that are least likely to use the supports that are available to them.

Other supports are available to NHS staff. Occupational health departments, often called health and well-being services, can support HCWs with a variety of mental and physical health problems that may be caused by or affect work; they can also advise on reasonable adjustments to help HCWs to return to and/or remain in work.^18^ A recent review of occupational health interventions across multiple sectors concluded that more work was needed to systematically assess their effectiveness.^19^ Many trusts also offer employee assistance programmes (EAPs), which provide free telephone or online short-term counselling to staff via external companies, as well as referrals to designated mental health teams for those with more serious mental ill health.^20^ However, there is a lack of robust evidence on the efficacy of EAPs. Good support from managers and colleagues can also have a positive impact on the mental health of HCWs,^21,22^ whereas a lack of support can have a detrimental effect.^23^ Given the key role of HCWs during the pandemic, there was an increased focus on their well-being; several other supports were promoted at both national and trust levels, including well-being hubs,^24^ free meals and parking,^16,22^ dedicated relaxation spaces,^25^ and mobile mental health and well-being apps.^26^ The evidence suggests that HCWs have had positive experiences with many of these supports,^16,22,24^ although most have not been formally evaluated. Many barriers to help-seeking exist for these supports, i.e. well-being hubs;^16,27,28^ however, we have yet to see work that has focused on predictors of access to these supports.

Whereas various supports are available to HCWs in the UK, the proportion of staff using them is unclear. In addition, although we understand many of the barriers to help-seeking among HCWs, evidence on demographic, occupational and psychosocial predictors is limited, in terms of both the number of studies and the supports included in them. We utilised data from a longitudinal study monitoring the mental health and well-being of NHS staff (from October 2020 to May 2023) to examine support use among a representative sample of clinical and non-clinical HCWs in England and to examine baseline predictors of access to these services. Using data collected at one of the three time points available to us, we explored whether the observed predictors of access to formal mental health support differed from those associated with access to a range of supports made available to HCWs, to identify potential inequalities within our sample and inform strategies to increase support uptake. Our research aims were to explore:

(a) self-reported use of mental health and well-being supports available to HCWs in England;

(b) baseline demographic, occupational and psychosocial predictors associated with use of formal mental health support at three follow-up data collection time points; and

(c) baseline demographic, occupational and psychosocial predictors associated with use of a range of supports compared with no support at one follow-up data collection time point.

## Method

### NHS CHECK and data collection

We analysed data from NHS CHECK, a longitudinal survey distributed to all HCWs (clinical and non-clinical), students and volunteers in 18 NHS trusts.^29^ A trust (a group of hospital, community and specialist care centres) operates as an independent organisation within the NHS in England, and the trusts included in this study were selected to offer diversity with respect to geographical location, setting (urban and rural), and type of trust (acute or mental health).^29^ Using workforce data provided by each trust, we could obtain accurate information about the population from which our sample was drawn, meaning that we could calculate response rates and sampling weights. The trusts circulated emails explaining and promoting the survey to all eligible HCWs. We also promoted the study via existing staff support teams or leads, chief nursing officers, medical directors, occupational health departments, trade union representatives and well-being hub users. NHS CHECK was discussed at team briefings, advertised via screen savers on trust computers, and included in trust newsletters, news items on trust intranet websites and closed social media groups.

Data for NHS CHECK were collected across four time points. Baseline data were collected between April 2020 and January 2021. Follow-up surveys were distributed to participants approximately 6 months (time 1; October 2020 and August 2021), 12 months (time 2; April 2021 and January 2022) and 32 months (time 3; February to May 2023) post-baseline. Responses across each time point were linked by participant ID number to enable longitudinal exploration. Informed consent was collected electronically before participation. The baseline NHS CHECK survey was completed by 22 092 HCWs (∼16% of the population). At 12 months, ∼1500 HCWs who had not completed the baseline or time 1 surveys were recruited; however, data for these HCWs were not included in the present work, as the lack of baseline data meant these participants could not be included in our predictor analyses. An overview of the NHS CHECK cohort can be found in a previous study exploring descriptive statistics for the full sample over all four time points.^30^ We followed the Strengthening the Reporting of Observational Studies in Epidemiology guideline for cohort studies.^31^

Ethical approval for the NHS CHECK study was granted by the Health Research Authority (reference: 20/HRA/2107, IRAS: 282686), as well as local trust research and development approval.

### Outcome: support use

**Fig. 1:**
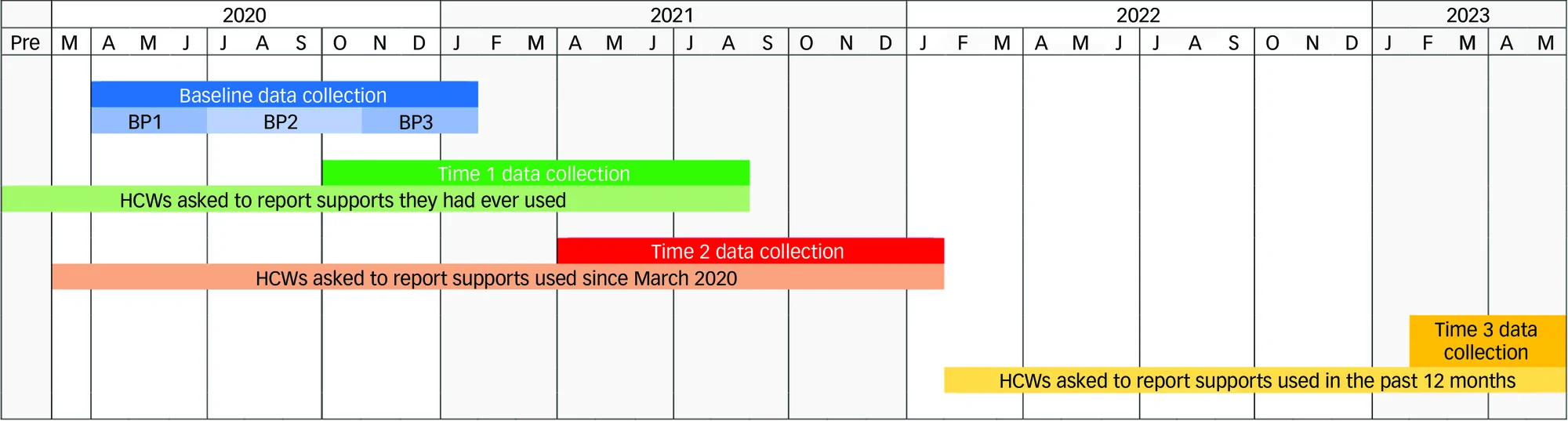
Timeline, with years and the first letter of each month, for the NHS CHECK data collection time points, along with the recall periods specified in the support services questions in each follow-up. ‘Pre’ indicates any time before March 2020, when the UK government began the first national lockdown because of COVID-19. ‘BP’ indicates the baseline burden period, i.e. periods of time during which numbers of cases were higher or lower, which may have affected reporting of baseline predictors (BP1 = April–June 2020, COVID-19 deaths = 53 389 (high burden); BP2 = July–October 2020, COVID-19 deaths = 7157 (low burden); BP3 = November 2020–March 2021, COVID-19 deaths = 88 211 (high burden)). BP, baseline predictor; NHS, National Health Service; HCWs, healthcare workers.

We collected data about support use in each of the follow-up surveys. See Fig. 1 for an overview of the data collection time points and the support recall periods for each. At time 1, participants were asked to ‘evaluate the national and local staff support offered to you’ and report whether they had ever accessed nationally offered supports (*n* = 8) and additional trust-specific supports (*n* = 88). The trust-specific supports were provided for staff locally, with each trust offering different supports, hence the large number in total (*n* = 96). These were identified through discussions with our patient and public involvement and engagement group and study contacts in each trust. We grouped the 96 supports into 7 categories (see Table 1 for examples of the 96 supports), through 3 discussions between the lead and co-authors, many of whom were also working in the NHS and had expertise in occupational health, psychiatry and mental health research.

**Table 1:** The 7 support categories devised by the research team using the 96 supports collected at time 1, with examples of the individual supports that were measured Table 1 long description.

Support category	Examples
Formal mental health support	Face-to-face, phone or online-based therapy provided in the clinic or ward, by a general practitioner or a community mental health team, in a well-being hub or in a private practice, or reported use of psychotropic medications
Occupational health	Engaging with a multidisciplinary occupational health team that supports healthcare workers with a variety of mental and physical health problems which may be caused by workplace factors or may affect workplace performance
Employee assistance programmes	Free telephone or online short-term counselling on professional and personal matters provided by external agencies and, for those with more serious mental ill health, signposting to designated mental health teams
Informal mental health support	Staff support phone and text lines, well-being apps, support or welfare services provided by a charity or spiritual leaders/chaplains
Workplace support	Virtual staff common rooms, leadership mentoring, support from managers, and team/peer support and reflective practice activities (i.e. Schwartz rounds and Team Time)
Practical support	Free or discounted food, parking, accommodation, services (such as childcare and insurance), salary advice and relaxation rooms
Information support	Information provided by management on self-care, additional supports, stress management, and briefings and guidance around COVID-19

At times 2 and 3, we asked HCWs whether they had accessed ‘mental health support’ from a general practitioner, NHS community mental health team, NHS talking therapies or a private mental healthcare team. We grouped these data under formal mental health supports. At time 2, we asked participants whether they had accessed these supports since March 2020, whereas at time 3, they were asked to report whether they had accessed them within the preceding 12-month period, ensuring that both measurements were unique. Participants may have accessed any of the other supports during these time points, but these were not measured in the time 2 or 3 surveys. There was also an overlap between times 1 and 2 (Fig. 1), and some HCWs may have reported use of the same support at both time points. Time 3 was distinct.

At each time point, we recoded responses from additional free-text questions asking what supports had been used in the respective periods into the corresponding support categories (time 1, 2838 responses; time 2, 676 responses; time 3, 422 responses). For analysis, we created eight outcome variables at time 1, with seven identifying staff who had accessed supports in each of the seven categories and the eighth indicating HCWs who had not accessed any support. At times 2 and 3, we had one outcome variable each; this identified HCWs who had utilised formal supports at each time point.

Noting similarities between the support categories at time 1 (i.e. that HCWs may have accessed therapy or counselling via occupational health, EAP, and formal and informal mental health supports), we decided to create an additional variable in which we regrouped the categories. This resulted in three distinct groups, defined as follows: 0 = never used any support, 1 = used any active support (i.e. occupational health, EAP, or formal or informal mental health support), and 2 = used passive support only (i.e. workplace, practical and information support). This variable was hierarchical, i.e. if HCWs had accessed both active and passive support, they were coded as using active support. We developed this variable using the authors’ expert opinion and knowledge of a similar hierarchical method used to group supports in previous research.^32^ It was used to identify HCWs who made direct, interpersonal and often structured engagement with another person or service to improve health and well-being (active support), as well as those who had used only less formal passive supports (e.g. sought managerial or peer support, read a health and well-being newsletter or received practical support) and those who had not used any support. This was considered as a supplementary analysis once we had started the formal analysis.

### Baseline predictor variables

We included baseline predictors based on team discussions and our knowledge of previous international and NHS CHECK studies to explore how demographic, occupational and psychosocial factors could influence the odds of accessing supports. These included participants’ sex, age, ethnicity, job role, relationship status, length of NHS service and contract type. We also included the 12-item General Health Questionnaire (GHQ-12) to screen for symptoms of CMDs, using the recommended cut-off score (≥4) to indicate probable caseness.^33^ To account for potential clustering in the sample, we also included two multilevel variables: the trust in which HCWs worked, and the burden period. We previously identified ‘burden periods’, i.e. the time at baseline when the reported numbers of COVID-19 cases and deaths were higher or lower, as these numbers are likely to have affected pressure on HCWs and may have influenced our predictors, particularly our mental health predictor^29,30^ (see Fig. 1 for an overview of these periods).

### Data analysis

We pre-registered a statistical analysis plan for the current study on the Open Science Framework;^34^ this paper covers Aim 3 of the plan. There were some deviations from the statistical analysis plan: we did not include baseline support data owing to concerns about its robustness (questions about supports used were free-text and had a low response rate); we used the GHQ-12 as the mental health predictor instead of other screening tools (i.e. nine-item Patient Health Questionnaire or Generalized Anxiety Disorder seven-item scale), as we found it was robust despite a small typographical error in one response option of one item.^35^ Specifically, we had erroneously replaced the ‘More so than usual’ response with ‘Not at all’ for item 8. To test whether this error could have affected our data, we conducted a double-blind randomised controlled trial, which found no significant differences in GHQ-12 score whether this error had been made, the same error had been made on item 1 instead of item 8, or no labelling error had been made. We excluded data about suicidal ideation owing to multi-collinearity with GHQ-12; and we did not include data about the helpfulness of each support at time 1, as this was collected for each of the 96 supports, and detailed analysis of these data was beyond the scope of this paper. Opportunities to examine helpfulness will be explored in future NHS CHECK research. The NHS CHECK cohort was weighted at baseline using a raking algorithm based on the age, sex, ethnicity and job role profile of the workforce at each trust to maximise representativeness.^29,30^ The data were also weighted to account for individual non-response at each time point of data collection. To complete the weighting, missing data were imputed via the fifth nearest neighbour algorithm. These imputed data were only used to complete weighting. Weighting was conducted using R version 4.0.2 for Windows (R Core team, Vienna, Austria; https://www.r-project.org/). Analysis for this study was conducted with Stata version 18 for Windows (Statacorp, College Station, Texas USA; https://www.stata.com/).

#### Aim 1

We began the formal analysis using descriptive statistics for the outcome and predictor variables. Specifically, we looked at the frequency and weighted percentage of complete and missing responses for each variable at each follow-up time. We did not employ any strategy to impute missing values, as our sample was adequate (approximately 87–89% of HCWs had complete data on all outcome and predictor variables at each time point). Before inclusion of predictors in regression modelling, we checked multi-collinearity between them using the variance inflation factor; we did not find evidence that multi-collinearity was a concern.

#### Aim 2

To explore predictors for accessing formal mental health supports, we conducted logistic regression modelling. Reported uses of supports at each follow-up time were used as outcomes for three regression models. We accounted for potential clustering by including trust and baseline burden period, resulting in multilevel models. In addition, we added a variable which accounted for reported use at previous time points. The odds ratios were adjusted for all predictors, and 95% confidence intervals are reported to demonstrate the significance of the associations between predictors and outcome.

#### Aim 3

To explore predictors for access to either active or passive versus no supports at time 1, we conducted multinomial logistic regression modelling. We conducted this analysis only for time 1, as data about the full list of 96 supports were not collected at times 2 and 3. We utilised Stata’s generalised structural equation modelling command with the *mlogit* link function. We fitted four models. The first did not include any predictors (null model), the second (model 1) included individual-level predictors, the third (model 2) included the community-level variables (i.e. trust and burden period) and the fourth (model 3) included all predictors. Model 3 had the lowest Akaike information criterion and Bayesian information criterion values (that is, the best statistical fit); it was therefore chosen as the most appropriate. This model is described later in this paper, whereas the remaining models are presented in Supplementary Table 1 available at https://doi.org/10.1192/bjo.2026.12108. For all models, relative risk ratios were adjusted for all predictors, and 95% confidence intervals are reported to demonstrate the significance of the association between predictors and outcome.

## Results

### Sample characteristics

Table 2 shows the demographic, occupational and psychosocial characteristics of the sample at each data collection time point. Across baseline and the three follow-up surveys, participants were mostly female (72.5–74.0%), White (74.5–77.4%), in a relationship (71.0–73.1%) and employed on a permanent contract (84.8–87.3%). Less than half of each subsample (42.4–47.9%) scored above the GHQ-12 cut-off at baseline, indicating probable CMDs. Around 10% of HCWs were doctors, with the remainder equally distributed among nursing and other clinical and non-clinical roles, and ∼60% of HCWs had worked in the NHS for 10 years or less. Variation across characteristics was mostly stable across the time points. We have provided a detailed overview of the NHS CHECK sample and variations between the baseline and follow-up time points in previous work.^30^

**Table 2:** Information on the demographic, occupational and psychosocial characteristics of the NHS staff who participated in each time point of data collection of the NHS CHECK longitudinal cohort^a^ Table 2 long description.

Characteristics	Baseline	Time 1	Time 2	Time 3
	(*N* = 22 092)	(*N* = 10 514)	(*N* = 10 429)	(*N* = 6382)
	*n* (%)	*n* (%)	*n* (%)	*n* (%)
Sex				
Female	17 726 (73.5)	8519 (74.0)	8449 (73.1)	5085 (72.5)
Male	4116 (25.4)	1934 (24.5)	1901 (24.8)	1236 (24.3)
Missing	250 (1.1)	61 (1.5)	79 (2.1)	61 (3.2)
Age, years				
30 and younger	4230 (21.0)	1610 (19.0)	1442 (19.0)	710 (16.3)
31–40	4848 (25.4)	2134 (24.3)	1952 (22.6)	1194 (23.4)
41–50	5522 (21.9)	2759 (21.2)	2818 (20.9)	1822 (22.1)
51–60	5147 (20.2)	2835 (19.7)	2965 (19.2)	1881 (18.7)
61 and older	1303 (6.6)	744 (6.0)	779 (5.6)	492 (5.3)
Missing	1042 (4.9)	432 (9.8)	473 (12.7)	283 (14.2)
Ethnicity				
White	18 738 (75.3)	9270 (77.4)	9269 (77.0)	5666 (74.5)
Black	971 (8.5)	332 (6.5)	334 (7.2)	193 (6.5)
Asian	1458 (11.9)	562 (11.3)	474 (10.5)	294 (12.3)
Mixed/multiple ethnic group	537 (1.2)	235 (1.3)	230 (1.5)	137 (1.5)
Other ethnic group	205 (2.3)	80 (2.8)	73 (2.7)	52 (3.5)
Missing	183 (0.8)	35 (0.7)	49 (1.1)	40 (1.7)
Job role				
Nurse	5652 (28.7)	2580 (28.1)	2640 (29.1)	1601 (29.1)
Doctor	1613 (10.0)	741 (10.7)	688 (9.5)	394 (9.1)
Other clinical	6654 (31.3)	3027 (30.8)	2952 (30.3)	1781 (30.4)
Non-clinical	8012 (29.3)	4139 (29.7)	4108 (30.1)	2570 (29.6)
Missing	161 (0.7)	27 (0.7)	41 (1.0)	36 (1.8)
Relationship status				
Single or divorced	5706 (27.0)	2618 (25.9)	2617 (27.1)	1595 (26.6)
Married or cohabitating	16 162 (72.0)	7841 (73.1)	7748 (71.6)	4733 (71.0)
Missing	224 (1.0)	55 (1.0)	64 (1.3)	54 (2.4)
Probable CMDs				
No (GHQ-12 score < 4)	9655 (44.1)	4653 (43.3)	5164 (48.1)	3048 (46.6)
Yes (GHQ-12 score ≥ 4)	10 694 (47.9)	4745 (45.8)	4383 (42.4)	2901 (46.1)
Missing	1743 (8.0)	1116 (10.9)	882 (9.5)	433 (7.3)
Contract type				
Permanent contract	18 820 (84.8)	9222 (86.6)	9229 (87.3)	5674 (87.0)
Non-permanent contract	3021 (14.1)	1266 (12.9)	1136 (11.7)	661 (11.4)
Missing	251 (1.1)	26 (0.5)	64 (1.0)	47 (1.6)
Length of NHS service				
Less than 2 years	7010 (31.9)	3023 (31.0)	2768 (29.3)	1606 (27.9)
3–10 years	7532 (33.4)	3610 (33.2)	3581 (33.9)	2145 (33.2)
11–19 years	4103 (18.1)	2103 (18.5)	2143 (18.3)	1399 (20.2)
20 years or more	2750 (11.9)	1506 (12.8)	1619 (13.1)	1029 (13.0)
Missing	697 (4.7)	272 (4.5)	318 (5.4)	203 (5.7)

HCWs, healthcare workers; CMDs, common mental disorders; GHQ-12, 12-item General Health Questionnaire; NHS, National Health Service.

a. The baseline survey was distributed between April 2020 and January 2021; the time 1 survey was distributed between October 2020 and August 2021; the time 2 survey was distributed between April 2021 and January 2022; and the time 3 survey was distributed between February and May 2023. All data were collected at baseline.

### Reported support use

Reported support use is displayed in Table 3. At time 1, practical supports were the most commonly used, with 58.3% of HCWs having used such supports, followed by informal mental health support (45.4%), information (29.9%), occupational health (25.2%), formal mental health support (22.6%) and workplace supports (21.0%). EAPs were the least commonly used support option (4.8%). At time 1, 58.6% said they had used an active support to improve their mental health, whereas 20.9% indicated that they had only used passive support, and 20.5% had never used any support. At time 2, 16.4% of HCWs said that they had accessed formal mental health support between March 2020 and January 2022, whereas at time 3, 20.8% of HCWs had done so between February 2022 and May 2023.

**Table 3:** Information on support usage at each NHS CHECK data collection time point^a^ Table 3 long description.

Support type	Time 1	Time 2	Time 3
	(*N* = 10 514)	(*N* = 10 429)	(*N* = 6382)
	*n* (%)	*n* (%)	*n* (%)
Formal mental health support			
Reported using	2524 (22.6)	1813 (16.4)	1379 (20.8)
Did not report using	7990 (77.4)	8617 (83.6)	5003 (79.2)
Occupational health			
Reported using	2571 (25.2)	–	–
Did not report using	7943 (74.8)	–	–
Employee assistance programmes			
Reported using	578 (4.8)	–	–
Did not report using	9936 (95.2)	–	–
Informal mental health support			
Reported using	4853 (45.4)	–	–
Did not report using	5661 (54.6)	–	–
Workplace support			
Reported using	2196 (21.0)	–	–
Did not report using	8318 (79.0)	–	–
Practical support			
Reported using	6074 (58.3)	–	–
Did not report using	4440 (41.7)	–	–
Information support			
Reported using	2798 (29.9)	–	–
Did not report using	7716 (70.1)	–	–
Type of support accessed^b^			
Reported using no support	2103 (20.5)	–	–
Reported use of any active support	6353 (58.6)	–	–
Reported use of passive support only	2058 (20.9)	–	–

NHS, National Health Service.

a. Time 1 data were collected between October 2020 and August 2021, and participants were asked about support used ever; time 2 data were collected between April 2021 and January 2022, and participants were asked about support used since March 2020; and time 3 data were collected between February and May 2023, and participants were asked about support used in the past 12 months; 3499 healthcare workers completed all three surveys.

b. Active support includes occupational health, employee assistance programmes, and formal and informal mental health support. Passive support includes workplace, practical and information support. Note. If healthcare workers reported using any active support, they are included in this category even if they also used passive supports.

### Predictors for using formal mental health support at times 1, 2 and 3

We conducted binary logistic regressions to explore predictors for access to formal mental health support at each time point (Table 4). Figure 1 outlines the time periods of service use for each time point. Across all time points, we found that HCWs who screened positive for probable CMDs at baseline reported between 1.46 and 2.40 higher odds of accessing these supports (time 1 adjusted odds ratio (aOR) = 2.40, 95% CI 2.03, 2.84; time 2 aOR = 1.99, 95% CI 1.68, 2.36; time 3 aOR = 1.46, 95% CI 1.25, 1.71). We also found that HCWs of Asian ethnicity reported between 0.44 and 0.56 lower odds of accessing these supports (time 1 aOR = 0.56, 95% CI 0.45, 0.71; time 2 aOR = 0.53, 95% CI 0.37, 0.76; time 3 aOR = 0.44, 95% CI 0.30, 0.65). Further, our findings indicated that HCWs who had previously accessed a formal mental health support had more than five times higher odds of accessing such support again at times 2 and 3 (time aOR = 5.02, 95% CI 4.19, 6.02; time aOR = 5.55, 95% CI 4.16, 7.40).

**Table 4:** Multilevel binary logistic regression models^a^ exploring predictors of access to formal mental health support at each NHS CHECK follow-up time point of data collection Table 4 long description.

Predictor	Odds of accessing a formal mental health support								
	Time 1, *n* = 9365			Time 2, *n* = 9113			Time 3, *n* = 5550		
	aOR	*P*-value	95% CI	aOR	*P*-value	95% CI	aOR	*P*-value	95% CI
Sex									
Female (ref.)	1.00	–	–	1.00	–	–	1.00	–	–
Male	0.90	0.300	0.73, 1.11	**0.66**	**0.007**	**0.49, 0.88**	**0.81**	**0.004**	**0.71, 0.93**
Age, years									
30 and younger (ref.)	1.00	–	–	1.00	–	–	1.00	–	–
31–40	1.10	0.339	0.90, 1.33	0.91	0.511	0.67, 1.23	0.98	0.877	0.76, 1.27
41–50	**0.78**	**0.025**	**0.64, 0.97**	**0.80**	**0.041**	**0.65, 0.99**	**0.72**	**0.041**	**0.53, 0.99**
51–60	**0.60**	**<0.001**	**0.49, 0.73**	**0.71**	**0.005**	**0.57, 0.89**	0.74	0.068	0.53, 1.03
61 and older	**0.53**	**0.006**	**0.35, 0.81**	**0.40**	**<0.001**	**0.26, 0.62**	**0.54**	**0.011**	**0.34, 0.85**
Ethnicity									
White (ref.)	1.00	–	–	1.00	–	–	1.00	–	–
Black	**0.59**	**0.002**	**0.44, 0.80**	0.81	0.325	0.52, 1.26	0.70	0.305	0.35, 1.42
Asian	**0.56**	**<0.001**	**0.45, 0.71**	**0.53**	**0.002**	**0.37, 0.76**	**0.44**	**<0.001**	**0.30, 0.65**
Mixed/multiple ethnic group	**0.76**	**0.002**	**0.65, 0.89**	0.93	0.750	0.59, 1.48	**1.55**	**0.012**	**1.12, 2.16**
Other ethnic group	0.56	0.115	0.27, 1.17	1.25	0.608	0.51, 3.07	0.90	0.809	0.36, 2.26
Job role									
Nurse (ref.)	1.00	–	–	1.00	–	–	1.00	–	–
Doctor	0.74	0.121	0.50, 1.09	0.95	0.811	0.58, 1.53	1.01	0.961	0.72, 1.41
Other clinical	0.91	0.374	0.77, 1.13	0.96	0.656	0.79, 1.16	**0.82**	**0.017**	**0.70, 0.96**
Non-clinical	0.92	0.516	0.70, 1.21	0.81	0.184	0.58, 1.12	0.99	0.945	0.80, 1.23
Relationship status									
Single or divorced (ref.)	1.00	–	–	1.00	–	–	1.00	–	–
Married or cohabitating	**0.75**	**0.001**	**0.65, 0.87**	0.86	0.116	0.72, 1.04	1.00	0.996	0.78, 1.29
Probable common mental disorders at baseline									
No (GHQ-12 score < 4; ref.)	1.00	–	–	1.00	–	–	1.00	–	–
Yes (GHQ-12 score ≥4)	**2.40**	**<0.001**	**2.03, 2.84**	**1.99**	**<0.001**	**1.68, 2.36**	**1.46**	**<0.001**	**1.25, 1.71**
Contract type									
Permanent contract (ref.)	1.00	–	–	1.00	–	–	1.00	–	–
Non-permanent contract	0.95	0.466	0.82, 1.10	1.15	0.140	0.95, 1.38	0.92	0.543	0.70, 1.22
Length of NHS service									
Less than 2 years (ref.)	1.00	–	–	1.00	–	–	1.00	–	–
3–10 years	1.08	0.161	0.97, 1.21	0.91	0.321	0.75, 1.10	0.87	0.222	0.69, 1.10
11–19 years	0.91	0.295	0.77, 1.09	0.84	0.070	0.70, 1.02	**0.72**	**0.029**	**0.54, 0.96**
20 years or more	**0.70**	**0.003**	**0.57, 0.87**	**0.67**	**0.014**	**0.50, 0.91**	0.62	0.063	0.37, 1.04
Accessed formal support before each time point^b^									
No (ref.)	–	–	–	1.00	–	–	1.00	–	–
Yes	–	–	–	**5.02**	**<0.001**	**4.19, 6.02**	**5.55**	**<0.001**	**4.16, 7.40**

aOR, adjusted odds ratio (adjusted for all variables in the table, as well as burden on the NHS when risk factor data were collected and trust); GHQ-12, 12-item General Health Questionnaire; NHS, National Health Service.

a. Baseline predictor data were collected between April 2020 and January 2021. Time 1 data were collected between October 2020 and August 2021, and participants were asked about support used ever; time 2 data were collected between April 2021 and January 2022, and participants were asked about support used since March 2020; and time 3 data were collected between February and May 2023, and participants were asked about support used in the past 12 months. Data were weighted on the basis of the age, sex, ethnicity and job role profile of the workforce at each trust, as well as non-response across time points of data collection, to maximise representativeness. Bold text indicates statistical significance according to the reported *P*-values. Outcomes were coded 0 = did not report use of formal mental health support; or 1 = did report use.

b. At time 1, healthcare workers were asked whether they had ever used formal mental health support, so we could not account for previous use of these services.

At time 1, we found that HCWs who were of mixed or multiple ethnicities had 0.76 lower odds of accessing formal supports (95% CI 0.65, 0.89), compared with 1.55 higher odds at time 3 (95% CI 1.12, 2.16). At times 2 and 3, male HCWs had between 0.66 and 0.81 lower odds of accessing these supports (time aOR = 0.66, 95% CI 0.49, 0.88; time 3 aOR = 0.81, 95% CI 0.71, 0.93). We also found that HCWs aged 41 years or older reported lower odds of accessing these supports across all three time points, except for the 51–60 age group at time 3 (see Table 4 for information about these associations).

### Predictors of use of active or passive support at time 1

We conducted a multinomial logistic regression to explore predictors of having accessed any active support or passive support only, compared with having never accessed any support by time 1 (Table 5). First, we found that male HCWs (adjusted relative risk ratio, aRRR = 0.65, 95% CI 0.59, 0.73) and those on a non-permanent contract (aRRR = 0.64, 95% CI 0.54, 0.75) had lower odds of accessing active support compared with those who had never accessed any support, whereas HCWs who screened for probable CMDs at baseline had greater odds of accessing active support (aRRR = 1.73, 95% CI 1.59, 1.87). We also found that HCWs of Asian ethnicity (aRRR = 0.57, 95% CI 0.40, 0.82) or aged over 41 years (41–50 age group aRRR = 0.75, 95% CI 0.61, 0.91; 51–60 age group aRRR = 0.60, 95% CI 0.45, 0.80; 61+ age group aRRR = 0.41, 95% CI 0.28, 0.60) had lower odds of accessing active support, whereas other clinical HCWs had higher odds (aRRR = 1.27, 95% CI 1.10, 1.47) of accessing active support. When comparing HCWs who had never accessed support with those who had accessed passive support only, we found that HCWs of Asian ethnicity (aRRR = 0.38, 95% CI 0.24, 0.61), HCWs aged 61 years and older (aRRR = 0.46, 95% CI 0.31, 0.70) and HCWs employed on non-permanent contracts (aRRR = 0.69, 95% CI 0.51, 0.92) had lower odds of accessing passive support.

**Table 5:** Multilevel multinomial logistic regression model exploring predictors for access to any active support or passive support only at time 1 (by August 2021; *n* = 9365)^a^ Table 5 long description.

Predictor	Compared with HCWs who used no support (*n* = 1907)					
	Accessed any active support, *n* = 5546			Accessed passive support only, *n* = 1912		
	aRRR	*P*-value	95% CI	aRRR	*P* -value	95% CI
Sex						
Female (ref.)	1.00	–	–	1.00	–	–
Male	**0.65**	**<0.001**	**0.59, 0.73**	0.90	0.337	[0.73, 1.11]
Age, years						
30 and younger (ref.)	1.00	–	–	1.00	–	–
31–40	1.11	0.219	0.94, 1.31	1.07	0.588	[0.83, 1.39]
41–50	**0.75**	**0.003**	**0.61, 0.91**	0.90	0.481	[0.67, 1.21]
51–60	**0.60**	**0.001**	**0.45, 0.80**	0.72	0.052	[0.51, 1.00]
61 and older	**0.41**	**<0.001**	**0.28, 0.60**	**0.46**	**<0.001**	**[0.31, 0.70]**
Ethnicity						
White (ref.)	1.00	–	–	1.00	–	–
Black	0.85	0.202	0.66, 1.09	0.90	0.509	0.65, 1.24
Asian	**0.57**	**0.003**	**0.40, 0.82**	**0.38**	**<0.001**	**0.24, 0.61**
Mixed or multiple ethnic group	1.03	0.939	0.54, 1.96	1.04	0.925	0.45, 2.40
Other ethnic group	0.76	0.568	0.30, 1.95	1.12	0.739	0.57, 2.20
Job role						
Nurse (ref.)	1.00	–	–	1.00	–	–
Doctor	0.74	0.150	0.49, 1.12	1.30	0.073	0.98, 1.74
Other clinical	**1.27**	**0.001**	**1.10, 1.47**	1.21	0.062	0.99, 1.47
Non-clinical	1.05	0.639	0.85, 1.29	1.19	0.106	0.96, 1.47
Relationship status						
Single or divorced (ref.)	1.00	–	–	1.00	–	–
Married or cohabitating	0.90	0.180	0.76, 1.05	0.98	0.879	0.80, 1.21
Probable common mental disorders at baseline						
No (GHQ-12 score <4; ref.)	1.00	–	–	1.00	–	–
Yes (GHQ-12 score ≥4)	**1.73**	**<0.001**	**1.59, 1.87**	0.98	0.744	0.86, 1.12
Contract type						
Permanent contract (ref.)	1.00	–	–	1.00	–	–
Non-permanent contract	**0.64**	**<0.001**	**0.54, 0.75**	**0.69**	**0.011**	**0.51, 0.92**
Length of NHS service						
Less than 2 years (ref.)	1.00	–	–	1.00	–	–
3–10 years	1.14	0.115	0.97, 1.35	1.19	0.095	0.97, 1.46
11–19 years	1.08	0.544	0.83, 1.41	1.22	0.101	0.96, 1.56
20 years or more	1.12	0.284	0.91, 1.38	1.29	0.053	1.00, 1.66

HCWs, healthcare workers; aRRR, adjusted relative risk ratio (adjusted for all variables in the table, as well as burden period and trust; GHQ-12, 12-item General Health Questionnaire; NHS, National Health Service.

a. Outcomes were coded 0 = used no support (reference group), 1 = used any active support, or 2 = used passive support only. If healthcare workers reported accessing any active support, they were included in the active support category regardless of whether they also accessed passive supports. Bold text indicates statistical significance, according to the reported *P*-value. Baseline predictor data were collected between April 2020 and January 2021, whereas data on support services ever used were collected between October 2020 and August 2021 (time 1). Data were weighted based on the age, sex, ethnicity and job role profile of the workforce at each trust, as well as non-response across time points of data collection.

## Discussion

### Formal mental health support

Approximately one-fifth of HCWs reported using formal mental health support at each time point. Time 1 had the highest proportion (22.6%); this was unsurprising, as this time point had the longest recall period (HCWs were asked whether they had ever accessed these supports before August 2021). We also found that 16.4% had accessed such support between March 2020 and January 2022 (time 2), whereas 20.8% had done so between February 2022 and May 2023 (time 3). These results are comparable with those of the Adult Psychiatric Morbidity Survey (APMS), which estimated that 15.7% of the UK population were in receipt of mental health treatment (psychotropic medication and/or psychological therapy) between March 2023 and July 2024.^36^ Although our estimates were slightly higher, the APMS asked participants whether they were receiving treatment at the time of the interview, whereas we asked whether they had used a support within a longer time period.

Promisingly, HCWs who reported symptoms of a CMD at baseline were more likely to have accessed formal support at follow-up than HCWs who had not reported symptoms. A similar finding was reported by the 2023/24 APMS.^36^ These findings indicate that many of those who may benefit most from support are accessing it. Approximately half of our baseline sample reported symptoms of a CMD. We have previously written about how self-report screening tools (such as the GHQ) overestimate CMDs and noted that we believe the true prevalence to be much lower (diagnostic interviews indicated that 21.5% of HCWs met the diagnostic criteria for a CMD).^9^ Although support use in this study fell below previous estimates that 28–30% of HCWs may experience symptoms of a CMD,^8^ it was consistent with our estimate of 21.5% of HCWs meeting criteria following assessment.^9^ In addition, at times 2 and 3, we found that HCWs who had previously accessed a similar service had more than five times the odds of using the service again, suggesting that they may have had positive experiences with the support they received, or that their difficulties were ongoing and they required continued support. Those who do not access formal mental health support are probably deterred by barriers to help-seeking such as stigma, negative career implications, hostile work environments, a lack of awareness or lack of availability of support, and lack of time to seek help.^15,16^ Not all HCWs need engage with these supports, although it is important that those who may need or want them are able to access them.^37^

### All supports

At time 1, we found that 58.6% of HCWs had ever used a form of active support, including occupational health, EAPs, or formal or informal mental health support. We grouped these together as we believe they have a common goal of directly improving HCW’s mental health (via therapy, counselling or other activities), and that HCWs are making active decisions to improve their own mental health by accessing them. Although it is positive that more than half of HCWs had engaged with at least one form of active support, it may also be concerning that so many perceived a need for these. The most frequently used active supports were informal mental health support (45.4%; i.e. staff phone and text lines, smartphone well-being apps, and welfare services provided by a charity or spiritual leaders/chaplains). These initiatives were often informal and easy to access, which probably increased their use. Research has also found that use of smartphone apps,^26^ for example, can lead to improvements in mental health and well-being among HCWs. Informal support should continue to be promoted as an appealing and easily accessible option, given that its flexibility may circumvent the aforementioned barriers to access to more formal support.^15,16^ We also found that 25.2% of HCWs had ever accessed occupational health, whereas only 4.8% had engaged with an EAP. EAPs were the least frequently used form of support, even though they were available in all but four of the trusts included in this study; the reasons for this are unclear, although they may be related to previously identified barriers.^15,16^ More work is needed to evaluate the effectiveness of occupational health services^19^ and EAPs to establish whether they are effective at improving outcomes for staff, and to explore experiences with them and potential barriers to access.

Practical supports were the most commonly used support category in our sample; these included free or discounted food, parking, accommodation or childcare. Almost two-thirds (58.3%) of the sample had used them before August 2021 (the end of our time 1 data collection period). Access to supportive information on self-care, additional supports, stress management and COVID-19 guidance was also common (29.9%). One-fifth of the sample had accessed workplace supports (21.0%) such as virtual staff common rooms, leadership mentoring, support from managers, and team support and reflective practice activities. Collectively, we refer to these as passive supports, as they are less formal and require lower levels of engagement than the active forms of support. Although there is evidence that many active supports can improve health and well-being,^12,13,18^ the impact of passive support is unclear. We are not aware of any research that has quantitatively examined the potential benefits of all these forms of support, although qualitative work has found that HCWs appreciated practical support and felt devalued and demoralised when it was taken away,^22^ and HCWs have reported positive experiences with reflective practices such as Schwartz rounds.^38^ It is unclear why more than 40% of HCWs in our sample did not access any passive support, although this may have been owing to unavailability in the trust, or a lack of awareness, or lack of time, need or desire to engage with such support.^16,22^ It is important to provide and promote these supports, as they may help to increase morale among HCWs.^22^ Further, 20.5% of the full time 1 sample said that they had never used any support. Again, although not every HCW may experience mental ill health, it is reasonable that HCWs should have access to passive or informal forms of support, and opportunities to provide and promote these should be explored. Importantly, we found that some staff were less likely to have used support, indicating potential inequalities with respect to access.

### Predictors of support use

Despite availability, many HCWs do not access supports, and the reasons for this are complex. Although many barriers to help-seeking have previously been reported,^15,16^ we identified several demographic, occupational and psychosocial predictors associated with not using these supports. We found that HCWs who were male, older (41+ years of age) or of Asian ethnicity had lower odds of accessing both formal mental health support and active support more broadly. HCWs of Asian ethnicity also had lower odds of accessing passive support. Although these associations are important, we have found that HCWs with these characteristics (particularly older age and male sex) also have lower odds of reporting symptoms of mental ill health across many NHS CHECK data collection time points.^30^ With this in mind, it is unsurprising that fewer males had accessed support; others have attributed similar findings to stereotypes that men should be stoic, self-reliant and unemotional.^39^ With respect to age, a review has found that older adults are more likely to hold negative cultural views about mental health, and that these views may reduce help-seeking;^40^ we believe that older HCWs may also be more likely to seek support from family or friends. HCWs of Asian ethnicity had lower odds of accessing support, which may be related to negative values about mental health and help-seeking held in many Asian cultures.^41^ In addition, a recent NHS staff survey found that experiences of discrimination from colleagues, patients and the public were common among staff from minoritised ethnic groups;^42^ this may have influenced their decisions regarding accessing support. Notably, the odds of accessing formal support were reversed among HCWs of mixed or multiple ethnicities between times 1 and 3; it is unclear why this occurred, although we acknowledge it may have been a spurious result due to multiple testing. In addition, HCWs employed on non-permanent contracts were less likely to use both active and passive support, although this was not observed when we looked only at formal mental health support. It may be that supports (especially informal mental health support, occupational health or EAPs) are not widely available to or promoted to many HCWs on fixed-term, training or bank/locum contracts, or that these staff do not have time to access them or believe that seeking support might jeopardise their position. Opportunities to promote support to non-permanent staff should be explored, given that the NHS heavily relies on such staff.^43^ Further work is needed to examine ways of encouraging support use among individuals within these groups.

### Strengths and limitations

The primary strength of this study was its longitudinal nature. This allowed us to examine support service usage across multiple time points and examine its predictors at times 2 and 3 (at time 1, participants were asked about supports ever used, so the data may pre-date baseline). In addition, using data provided by each trust, we weighted our sample by sex, age, ethnicity and job role to better represent the population from which participants were drawn. The sample was also weighted to account for individual non-response at each survey time point. Last, our list of 96 national and trust-level supports at time 1 helped us to obtain a comprehensive overview of all supports available to NHS staff in the studied trusts during the pandemic. For this reason, we believe our results are likely to be reflective of the availability and use of support across the wider NHS.

Our study had its limitations. First, our data relied on self-reporting; hence, there may have been bias in the responses. This may have been the case particularly for the time 1 survey, which had the longest recall period, asking HCWs whether they had ever accessed any of the supports. It may also have been a factor for the 20.5% of HCWs who did not report any support at time 1, as they may have forgotten or not reported support they had accessed years previously. Second, we note that the ‘Asian’ ethnic category included HCWs from a broad range of cultures (including Indian, Pakistani, Bangladeshi, Chinese, Arab or any other Asian background), and that drawing finite conclusions from this group may be difficult. Finally, although we knew whether HCWs had accessed any of the supports, we did not know how many times they had accessed each support, what they thought about the support they received, or potential barriers to access that may have affected their level of service utilisation. Similarly, we did not have data regarding the efficacy of these supports. These aspects, which are likely to hold important contextual factors that influenced HCWs’ engagement with the support available to them, were beyond the scope of this paper. However, we are currently examining ways to analyse the free-text data that were collected at times 2 and 3, in which HCWs reported on their experiences with formal mental health support.

### Future work and implications

By the end of our time 1 follow-up (ending August 2021), 79.5% of HCWs in our study had accessed at least one of the supports available to them, which meant that 20.5% of the sample had not accessed any support. Practical and informal mental health support were used by most HCWs, and we encourage future studies to examine the potential benefits of these; we believe that the former may increase staff morale, whereas the latter could improve HCWs’ mental health and should be promoted to circumvent known barriers to access to formal mental health support. EAPs were scarcely used, and the reasons for this were unclear. Future work should explore why HCWs who are male, older, employed on non-permanent contracts or of Asian ethnicity were less likely to access support. Ensuring that NHS staff feel supported, valued and able to complete their work should be a priority.

## Supporting information

10.1192/bjo.2026.12108.sm001 Dempsey et al. supplementary material Dempsey et al. supplementary material

## Data Availability

Owing to research ethics committee restrictions, the data-set is not publicly available. Requests to access the de-identified data-set can be made to the NHS CHECK team at nhscheck@kcl.ac.uk.

## Table 1 Long description

The table categorizes staff support into seven categories and provides examples of each. It has seven rows and two columns. The first column lists the support categories, and the second column provides examples of the supports within each category. The support categories are: Formal mental health support, Occupational health, Employee assistance programmes, Informal mental health support, Workplace support, Practical support, and Information support. Examples include face-to-face therapy, multidisciplinary occupational health teams, telephone counseling, staff support phone lines, virtual staff common rooms, free or discounted food, and information on self-care and COVID-19 briefings.

Navigate back to Table 1.

## Table 2 Long description

The table presents demographic, occupational, and psychosocial characteristics of NHS staff at four different time points: Baseline, Time 1, Time 2, and Time 3. The table has 11 rows and 8 columns, including headers. The columns are labeled as Baseline (N = 22 092), Time 1 (N = 10 514), Time 2 (N = 10 429), and Time 3 (N = 6382), with each column showing the number and percentage of participants for various characteristics. The rows are labeled with characteristics such as Sex, Age, Ethnicity, Job role, Relationship status, Probable CMDs, Contract type, and Length of NHS service. Each row provides the count and percentage of participants for each characteristic at each time point. For example, the Sex row shows the distribution of female, male, and missing data across the time points. The Age row shows the distribution of different age groups. The Ethnicity row shows the distribution of different ethnic groups. The Job role row shows the distribution of different job roles. The Relationship status row shows the distribution of single or divorced and married or cohabitating participants. The Probable CMDs row shows the distribution of participants with and without probable common mental disorders. The Contract type row shows the distribution of participants with permanent and non-permanent contracts. The Length of NHS service row shows the distribution of participants based on their length of service in the NHS.

Navigate back to Table 2.

## Table 3 Long description

A table comparing support usage among NHS staff at three different time points. The table has 10 rows and 9 columns. Column headers are Support type, Time 1 (N = 10 514) n (%), Time 2 (N = 10 429) n (%), and Time 3 (N = 6382) n (%). Row labels include Formal mental health support, Occupational health, Employee assistance programmes, Informal mental health support, Workplace support, Practical support, Information support, and Type of support accessed. Each row shows the number and percentage of staff reporting the use of different types of support at each time point. For example, at Time 1, 2524 (22.6%) reported using formal mental health support, while at Time 3, 1379 (20.8%) reported using it. The table also includes data on the reported use of no support, active support, and passive support.

Navigate back to Table 3.

## Table 4 Long description

The table presents the results of binary logistic regressions exploring predictors for access to formal mental health support at three different time points. It includes data for 9365 participants at Time 1, 9113 at Time 2, and 5550 at Time 3. The table is organized into columns for adjusted odds ratio (aOR), P-value, and 95% confidence interval (CI) for each predictor across the three time points. Predictors include sex, age, ethnicity, job role, relationship status, probable common mental disorders at baseline, contract type, length of NHS service, and previous access to formal mental health support. Each row provides the aOR, P-value, and 95% CI for each predictor at each time point. Notable trends include higher odds of accessing support for those with probable common mental disorders at baseline and those who had previously accessed support. Additionally, certain ethnic groups and older age groups show lower odds of accessing support.

Navigate back to Table 4.

## Table 5 Long description

A table comparing predictors for accessing any active support or passive support only among healthcare workers. The table has 13 rows and 10 columns. Column headers are Predictor, aRRR, P-value, 95% CI for those who accessed any active support, and aRRR, P-value, 95% CI for those who accessed passive support only. Row labels include Sex, Age, Ethnicity, Job role, Relationship status, Probable common mental disorders at baseline, Contract type, and Length of NHS service. Each row provides adjusted relative risk ratios (aRRR), P-values, and 95% confidence intervals (CI) for different predictors. Notable trends include lower odds of accessing active support for male healthcare workers and those on non-permanent contracts, and higher odds for those with probable common mental disorders at baseline. Healthcare workers of Asian ethnicity and older age groups also had lower odds of accessing active support.

Navigate back to Table 5.
