# Supplementary material for: Prevalence and predictors of use of mental health and well-being support among healthcare workers in England: findings from NHS CHECK
Source: BJPsych Open. 2026 Oct 9;12(6):e253. doi: 10.1192/bjo.2026.12108 (PMC13748683; doi:10.1192/bjo.2026.12108)
Supplement: Supplementary file 1 [file S2056472426121085sup001.docx]

**Supplementary Table 1.** Multi-level multinomial logistic regression models ^a^ exploring predictors for accessing passive support only or accessing no support at NHS CHECK 6-month follow-up (*n* = 9,365). Four models were fitted to examine if the multi-level data were an appropriate fit for the analysis.

|  |  | **Model 1** | | | | **Model 2** | | | | **Model 3** | | | |
| --- | --- | --- | --- | --- | --- | --- | --- | --- | --- | --- | --- | --- | --- |
|  | **Null Model** ^b^ | **Individual-level predictors only** | | | | **Community-level predictors only** | | | | **Full model with all predictors** | | | |
|  |  | **Compared to HCWs who used no support (*n* = 1,907), risk of:** | | | | | | | | | | | |
|  |  | **Accessing any active support,**  ***n* = 5,546** | | **Accessing passive support only,**  ***n* = 1,912** | | **Accessing any active support,**  ***n* = 5,546** | | **Accessing passive support only,**  ***n* = 1,912** | | **Accessing any active support,**  ***n* = 5,546** | | **Accessing passive support only,**  ***n* = 1,912** | |
|  |  | **aRRR** | **95% CI** | **aRRR** | **95% CI** | **aRRR** | **95% CI** | **aRRR** | **95% CI** | **aRRR** | **95% CI** | **aRRR** | **95% CI** |
| **Sex** |  |  |  |  |  |  |  |  |  |  |  |  |  |
| Female (Ref) |  | 1.00 | – | 1.00 | – |  |  |  |  | 1.00 | – | 1.00 | – |
| Male |  | **0.65** | **[0.55, 0.78]** | 0.89 | [0.72, 1.11] |  |  |  |  | **0.65** | **[0.59, 0.73]** | 0.90 | [0.73, 1.11] |
| **Age (years)** |  |  |  |  |  |  |  |  |  |  |  |  |  |
| 30 and younger (Ref) |  | 1.00 | – | 1.00 | – |  |  |  |  | 1.00 | – | 1.00 | – |
| 31–40 |  | 1.14 | [0.87, 1.49] | 0.99 | [0.72, 1.36] |  |  |  |  | 1.11 | [0.94, 1.31] | 1.07 | [0.83, 1.39] |
| 41–50 |  | **0.74** | **[0.56, 0.97]** | 0.80 | [0.58, 1.10] |  |  |  |  | **0.75** | **[0.61, 0.91]** | 0.90 | [0.67, 1.21] |
| 51–60 |  | **0.59** | **[0.45, 0.76]** | **0.62** | **[0.45, 0.85]** |  |  |  |  | **0.60** | **[0.45, 0.80]** | 0.72 | [0.51, 1.00] |
| 61 and older |  | **0.41** | **[0.29, 0.57]** | **0.41** | **[0.27, 0.62]** |  |  |  |  | **0.41** | **[0.28, 0.60]** | **0.46** | **[0.31, 0.70]** |
| **Ethnicity** |  |  |  |  |  |  |  |  |  |  |  |  |  |
| White (Ref) |  | 1.00 | – | 1.00 | – |  |  |  |  | 1.00 | – | 1.00 | – |
| Black |  | 0.99 | [0.70, 1.42] | 0.87 | [0.55, 1.38] |  |  |  |  | 0.85 | [0.66, 1.09] | 0.90 | [0.65, 1.24] |
| Asian |  | **0.63** | **[0.46, 0.87]** | **0.45** | **[0.30, 0.67]** |  |  |  |  | **0.57** | **[0.40, 0.82]** | **0.38** | **[0.24, 0.61]** |
| Mixed/Multiple ethnic group |  | 1.16 | [0.68, 1.98] | 1.04 | [0.53, 2.06] |  |  |  |  | 1.03 | [0.54, 1.96] | 1.04 | [0.45, 2.40] |
| Other ethnic group |  | 0.89 | [0.43, 1.81] | 1.27 | [0.55, 2.92] |  |  |  |  | 0.76 | [0.30, 1.95] | 1.12 | [0.57, 2.20] |
| **Job Role** |  |  |  |  |  |  |  |  |  |  |  |  |  |
| Nurse (Ref) |  | 1.00 | – | 1.00 | – |  |  |  |  | 1.00 | – | 1.00 | – |
| Doctor |  | 0.78 | [0.58, 1.05] | 1.34 | [0.94, 1.91] |  |  |  |  | 0.74 | [0.49, 1.12] | 1.30 | [0.98, 1.74] |
| Other clinical |  | 1.17 | [0.94, 1.45] | 1.11 | [0.86, 1.44] |  |  |  |  | **1.27** | **[1.10, 1.47]** | 1.21 | [0.99, 1.47] |
| Non-clinical |  | 1.04 | [0.86, 1.25] | 1.15 | [0.91, 1.46] |  |  |  |  | 1.05 | [0.85, 1.29] | 1.19 | [0.96, 1.47] |
| **Relationship Status** |  |  |  |  |  |  |  |  |  |  |  |  |  |
| Single/Divorced (Ref) |  | 1.00 | – | 1.00 | – |  |  |  |  | 1.00 | – | 1.00 | – |
| Married/Cohabitating |  | 0.87 | [0.72, 1.04] | 1.00 | [0.81, 1.25] |  |  |  |  | 0.90 | [0.76, 1.05] | 0.98 | [0.80, 1.21] |
| **Probable Common Mental Disorders at baseline** | | | | | |  |  |  |  |  |  |  |  |
| No (GHQ-12 score <4; Ref) |  | 1.00 | – | 1.00 | – |  |  |  |  | 1.00 | – | 1.00 | – |
| Yes (GHQ-12 score ≥4) |  | **1.73** | **[1.48, 2.02]** | 0.98 | [0.81, 1.18] |  |  |  |  | **1.73** | **[1.59, 1.87]** | 0.98 | [0.86, 1.12] |
| **Contract type** |  |  |  |  |  |  |  |  |  |  |  |  |  |
| Permanent contract (Ref) |  | 1.00 | – | 1.00 | – |  |  |  |  | 1.00 | – | 1.00 | – |
| Non-permanent contract |  | **0.64** | **[0.50, 0.81]** | **0.67** | **[0.49, 0.90]** |  |  |  |  | **0.64** | **[0.54, 0.75]** | **0.69** | **[0.51, 0.92]** |
| **Length of NHS Service** |  |  |  |  |  |  |  |  |  |  |  |  |  |
| Less than 2 years (Ref) |  | 1.00 | – | 1.00 | – |  |  |  |  | 1.00 | – | 1.00 | – |
| 3 – 10 years |  | 1.16 | [0.95, 1.42] | 1.27 | [1.00, 1.61] |  |  |  |  | 1.14 | [0.97, 1.35] | 1.19 | [0.97, 1.46] |
| 11 – 19 years |  | 1.11 | [0.87, 1.41] | **1.38** | **[1.03, 1.85]** |  |  |  |  | 1.08 | [0.83, 1.41] | 1.22 | [0.96, 1.56] |
| 20 years or more |  | 1.13 | [0.87, 1.47] | **1.51** | **[1.11, 2.05]** |  |  |  |  | 1.12 | [0.91, 1.38] | 1.29 | [1.00, 1.66] |
| **Burden on NHS at Baseline** ^c^ |  |  |  |  |  |  |  |  |  |  |  |  |  |
| Burden period 1 (High; Ref) |  |  |  |  |  | 1.00 | – | 1.00 | – | 1.00 | – | 1.00 | – |
| Burden period 2 (Low) |  |  |  |  |  | **0.88** | **[0.80, 0.98]** | **0.82** | **[0.69, 0.98]** | 1.01 | [0.85, 1.21] | 0.89 | [0.70, 1.12] |
| Burden period 3 (High) |  |  |  |  |  | 0.96 | [0.81, 1.14] | **0.79** | **[0.66, 0.96]** | 1.08 | [0.83, 1.41] | 0.89 | [0.73, 1.09] |
| **NHS Trust** |  |  |  |  |  |  |  |  |  |  |  |  |  |
|  |  |  |  |  |  | 1.00 | – | 1.00 | – | 1.00 | – | 1.00 | – |
|  |  |  |  |  |  | 2.72 | Cons | 2.72 | Cons | 2.72 | Cons | 2.72 | Cons |
| **AIC** | 260963.1 | 203196.6 | | | | 256177.1 | | | | 199452.6 | | | |
| **BIC** | 260977.6 | 203468.1 | | | | 256242.4 | | | | 199566.9 | | | |
| aRRR = Relative Risk Ratio, adjusted for all variables in the table, as well as burden period and Trust; 95% CI = 95% Confidence Intervals; GHQ-12 = General Health Questionnaire; NHS = National Health Service; AIC = Akaike information criterion; BIC = Bayesian information criterion. Baseline predictor data were collected between April 2020 and January 2021, while data on support services ever used were collected between October 2020 to August 2021 (Time 1). Data were weighted based on the age, sex, ethnicity, and job role profile of the workforce at each Trust, as well as non-response across timepoints of data collection.  ^a^ Outcome was coded 0 = used no support (reference group), 1 = used any active support, and 2 = used passive support only. Note. If HCWs reported accessing any active support, they are included in this category regardless of whether they also accessed passive supports.  ^b^ The null model was run with only the outcome variable (type of support accessed) and did not include any predictor variables. As there were no predictor variables, there are no statistics to display, only the AIC and BIC, which demonstrate statistical fit of the model.  ^c^ These are periods of time where cases were higher or lower and which may have impacted reporting of baseline predictors (BP1 = April-June 2020, COVID-19 deaths = 53,389 (high burden); BP2 = July-October 2020, COVID-19 deaths = 7,157 (low burden); BP3 = November 2020-March 2021, COVID-19 deaths = 88,211 (high burden)) | | | | | | | | | | | | | |
